# The Therapeutic Effect of 1,8-Cineol on Pathogenic Bacteria Species Present in Chronic Rhinosinusitis

**DOI:** 10.3389/fmicb.2019.02325

**Published:** 2019-10-22

**Authors:** Matthias Schürmann, Felix Oppel, Martin Gottschalk, Björn Büker, Christian Andreas Jantos, Cornelius Knabbe, Andreas Hütten, Barbara Kaltschmidt, Christian Kaltschmidt, Holger Sudhoff

**Affiliations:** ^1^Department of Otolaryngology, Head and Neck Surgery, Klinikum Bielefeld, Bielefeld, Germany; ^2^Thin Films and Physics of Nanostructures, Faculty of Physics, Bielefeld University, Bielefeld, Germany; ^3^Institute of Laboratory Medicine and Microbiology, Bielefeld, Germany; ^4^Institute for Laboratory and Transfusion Medicine, Heart and Diabetes Center North Rhine-Westphalia, Bad Oeynhausen, Germany; ^5^Molecular Neurobiology, Faculty of Biology, Bielefeld University, Bielefeld, Germany; ^6^Department of Cell Biology, Faculty of Biology, Bielefeld University, Bielefeld, Germany

**Keywords:** biofilm, chronic rhinosinusitis, *S. aureus*, host model system, 1, 8-Cineol

## Abstract

Chronic rhinosinusitis (CRS) is marked by an inflamed mucosa of sinuses and is accompanied by a significantly reduced quality of live. Since no guidelines for the treatment of CRS are available, long lasting clinical histories with health care costs adding up to dozens of billion $ annually are caused by CRS. The progression of CRS is often induced by bacterial infections and/or a shift in microbiome as well as biofilm formation. The exact microbiome alterations are still unclear and the impenetrable biofilm renders the treatment with common antibiotics ineffective. This study focuses on characterizing the microbiome changes in CRS and investigating the inhibition of biofilm growth by 1,8-Cineol, a small, non-polar and hence biofilm penetrating molecule with known antimicrobial potential. We performed MALDI-TOF MS based characterization of the microbiomes of healthy individuals and CRS patients (*n* = 50). The microbiome in our test group was shifted to pathogens (*Staphylococcus aureus*, *Escherichia coli*, and *Moraxella catarrhalis*). In contrast to published studies, solely based on cell culture techniques, we could not verify the abundance of *Pseudomonas aeruginosa* in CRS. The inhibition of bacterial proliferation and biofilm growth by 1,8-Cineol was measured for these three pathogens. Interestingly, *S. aureus*, the most prominent germ in CRS, showed a biofilm inhibition not simply correlated to its inhibition of proliferation. RT-qPCR confirmed that this was due to the downregulations of major key players in biofilm generation (agrA, SarA and σ^B^) by 1,8-Cineol. Furthermore we verified this high biofilm inhibition potential in a model host system consisting out of *S. aureus* biofilm grown on mature respiratory epithelium. A second host model, comprising organotypic slices, was utilized to investigate the reaction of the innate immune system present in the nasal mucosa upon biofilm formation and treatment with 1,8-Cineol. Interestingly *Staphylococcus epidermidis*, the cause of very common catheter infections, possesses a biofilm generation pathway very similar to *S. aureus* and might be treatable in a similar fashion. The two presented *in vitro* model systems might be transferred to combinations of every biofilm forming bacterial with most kind of epithelium and mucosa.

## Introduction

The chronic rhinosinusitis (CRS) is defined as a symptomatic inflammation of the nasal and sinus mucosa with persistent symptoms lasting beyond 12 weeks with a prevalence of around 6% in Europe (Hastan et al., 2011). Besides the inflammation most patients suffering from CRS exhibit nasal obstruction and facial pressure further more nasal discharge and hyposmia are very common symptoms. In addition about 25–30% of CRS patients develop nasal polyps (CRSwNP) (Stevens et al., 2016). In most cases physicians treat the symptoms with a combination of steroids (Joe et al., 2008), nasal irrigations (Bachmann et al., 2000) and antibiotics, when purulence is identified. Additionally decongestants, mucolytics, antihistamines and leukotriene Inhibitors (Suh and Kennedy, 2011) are applied with the aim to reduce the inflammation, controlling the infection and promoting the mucociliary clearance. As ultima ratio, sinus surgery is designated for patients with persistent symptoms despite a maximal pharmacologic therapy. Unfortunately, up to now there are no existing guidelines to treat CRS and its cause. This often results in a long lasting course of disease with a significant reduced quality of life for patients. Moreover, CRS related health care expenses in the United States are estimated to add up to a total of 8.6 billion $ annually (Bhattacharyya, 2011).

Even though scientific endeavors have expanded the knowledge about the cause of CRS, the exact etiology of CRS is still unknown. Most likely CRS is caused by multiple factor related to the host as well as to their environment (Kennedy, 2004). Assuredly, a disturbance in microbiome composition inside the nasal cavity takes place during CRS development. Unfortunately, many of these undertaken studies dealing with this topic are less methodologically sound (e.g., solely culture based). Hence, it is rather difficult to gain a consistent conclusion on the bacterial changes during the development and progression of CRS (reviewed in Mahdavinia et al., 2016). To gather more data regarding this topic we want to study the bacterial composition of the nasal microbiome of CRS in a central European region by MALD-TOF and compare it to a healthy control group. Anyhow, it is certain that *Staphylococcus aureus* is very abundant in the nasal cavity of CRS patients with prevalence between 40% (Niederfuhr et al., 2008) and 68% (Feazel et al., 2012). More detailed investigations revealed, that an increased colonization rate of *S. aureus* was detected in patients with CRSwNP (64%) compared to patients without nasal polyps (27%) (Van Zele et al., 2004).

Unfortunately, around 50% to 80% of the bacterial colonization in CRS is manifested in form of a biofilm (Sanclement et al., 2005; Sanderson et al., 2006; Singh et al., 2015) while no or very little biofilm could be detected in the healthy control groups (Foreman et al., 2009; Singh et al., 2015, respectively). Especially noteworthy biofilm formation of *S. aureus* is correlated with a poor development of the disease in CRS patients (Bendouah et al., 2006). This is mainly due to the fact, that the sessile bacteria are well protected from antibiotic treatments by the impenetrably of the biofilm matrix composed out of different extracellular polymeric substances (EPS) (Jefferson et al., 2005; reviewed in Hall and Mah, 2017). The EPS auf *S. aureus* is composed out of partly deacetylated poly-β(1-6)-N-acetylglucosamine (PIA) (Valle et al., 2003), extracellular-DNA (Rice et al., 2007), proteins, and amyloid fibrils (Schwartz et al., 2012) its composition changes upon environmental stress factors (Tango et al., 2018) to protect the sessile bacteria against harmful influences. Even though many studies revealed the main switches for the development and maturation of biofilm formation of *S. aureus*, namely agrA, SarA and σ^B^ (reviewed in Archer et al., 2011), there is no effective pharmaceutical treatment to inhibit the biofilm growth of *S. aureus*.

An interesting alternative to common antibiotics are essential oil which consist out of small non-polar molecules able to penetrate the cell wall and the cell membrane of prokaryotic cells. Their mode of action (reviewed in Bakkali et al., 2008; Nazzaro et al., 2013; Swamy et al., 2016) is mainly based on the disruption of the layered polysaccharides and phospholipids. This will permeabilize the membranes, which is associated with loss of e.g., potassium ions in *Escherichia coli* (Cox et al., 1998) and *S. aureus* (Cox et al., 2000). The reduction of the membrane potential and the collapse of the proton pump leads to the depletion of the ATP pool. Beyond this various intracellular effects reducing the viability of prokaryotes on different levels (reviewed in Bakkali et al., 2008; Swamy et al., 2016).

Studies on the antimicrobial effect of different essential oils on *S. aureus* biofilms showed that the concentration necessary to effectively inhibit biofilm growth ranges from 0.3–1.3 mg/ml (Nostro et al., 2007). Most important in this context is the high diffusion coefficient of the small non-polar components of essential oils making them superior to common antibiotics in terms of biofilm penetration potential. Hence, essential oils were shown to inhibit and eradicate *S. aureus* biofilms with a higher efficiency when compared to common antibiotics (Kavanaugh and Ribbeck, 2012). An interesting molecule in this context is 1,8-Cineol a main component of the essential eucalyptus oil. It is known to inhibit growths of *S. aureus* with a minimum inhibitory concentration around 5 mg/ml (Carson et al., 2002; Trombetta et al., 2005; Honorio et al., 2015). Apart from its antibacterial action this pharmaceutical is of particular interest for the treatment of CRS, due to its known anti-inflammatory properties in airway disease (reviewed in Juergens, 2014 and for reducing mucus production Sudhoff et al., 2015). Therefore, we investigated the antibacterial and biofilm inhibitory potential of 1,8-Cineol in respect to the pathogenic bacteria detected in CRS. Furthermore, in this study we demonstrate biofilm inhibitory and anti-inflammatory capabilities of 1,8-Cineol in two independent *in vitro* host model system of CRS.

## Materials and Methods

### Microbiological Analysis of Patients Samples

Nasal swaps from the middle meatus where taken from patients treated for CRS or patients treated for ENT related illnesses without any nasal symptoms (*n* = 50). Specimens were obtained after informed written consent according to local and international guidelines (Bezirksregierung Detmold/Münster/Ethical Cl. number 2012-015-f-S). The non-charcoal-containing swabs (UNI-TER, MEUS s.r.l, Italy) were immediately hauled by an licensed company. At the clinical microbiological laboratory of the *Institut für Laboratoriumsmedizin, Mikrobiologie und Hygiene at the Evangelisches Klinikum Bethel* the specimens were streaked out onto Columbia agar with 5% sheep blood, chocolate- and MacConkey-Agar (*n* = 50). Agar plates were incubated at 35°C in an atmosphere containing 5% CO_2_ for 48 h. Plates were examined for growth at 24 and 48 h. and semi quantitative analysis of each colony type was performed. Identification of grown colonies was carried out by the use of a MALDI-TOF MS instrument (Bruker, Bremen, Germany). Samples were also cultivated on Baird-Parker Agar to selectively isolate staphylococcus species. The isolated germs were again analyzed by MALDI-TOF MS in the *Herz- und Diabeteszentrum NRW* (*n* = 17).

### Cell Culture

For bacterial cell culture isolated strains of *S. aureus*, *Moraxella catarrhalis* and *E. coli* were grown in brain heart infusion (BHI) overnight at 37°C with shaking at 250 rpm (orbital shaker incubator ES-20; Biosan, Latvia). The overnight culture was cryopreserved in BHI supplemented with 20% Glycerol and stored at −80°C. For final experiments a small amount of these cryostocks were transferred into 50 ml BHI and incubated overnight at 37°C with shaking at 250 rpm.

For culture of respiratory epithelium nasal mucosa was utilized, which was obtained during nasal surgery of CRS patients, after informed written consent according to local and international guidelines (Bezirksregierung Detmold/Münster). Immediately after removal the tissue was stored on ice and transported to the cell culture lab. After removing excess connective tissue and clotted blot the mucosa was chopped into small pieces (approx. 2 mm^3^) and digested with collagenase (0.375 U/ml in PBS, NB4; SERVA Electrophoresis GmbH, Germany) at 37°C for 2 h. If necessary, erythrocyte lysis buffer (155 mM NH_4_Cl, 10 mM KHCO_3_, 0,1 mM EDTA @ pH 7.3) was applied after centrifugation of the suspension and decantation of the collagenase. Subsequently, the pelleted cells were resuspended in PneumaCult^TM^-Ex Plus media (STEMCELL Technologies Inc., Canada) and cultivated for 3–7 days in T25 cell culture flask (Sarstedt, Germany). During this time the media was changed every second day. Following this pre-cultivation, the cell were detached with Accutase (Capricorn, Germany), seeded with a density of 10^5^ cells/cm^2^ in cell culture inserts (MCHT12H48; Merck Millipore, Germany) and incubated at 37°C with 5% CO_2_. After 2–3 days of culture, the cells reached confluence and were raised to the air-liquid interface simultaneously the media was changed to PneumaCult^TM^-ALI Medium (STEMCELL Technologies Inc., Canada). Subsequently the media was changed every second day. After 14 days of maturation the cells began to produce mucus. To remove the mucus the apical chamber was washed with PBS during every media change. After 21 days of maturation the cell showed morphological features of respiratory epithelium e.g., cilia and were further utilized for experiments.

To cultivate organotypic nasal slices the tissue was cut in a plane parallel to the epithelial layer in 400 μm thick slices (McIlwain tissue chopper, Ted Pella, United States) and placed onto cell culture insert (MCHT12H48; Merck Millipore, Germany) with the epithelial layer facing upward. The slice-containing membranes were kept at the air-liquid interface with PneumaCult^TM^-ALI Medium (STEMCELL Technologies Inc., Canada) filling the basal camber and incubated at 37°C at 5% CO_2_. Every second day the medium was changed and the slice was gently washed with PBS. The slices were utilized in further experiments between the 6th and 8th day in culture.

### Determination of MIC

To determine the minimal inhibitory concentration (MIC) of 1,8-Cineol on planktonic bacteria a macrodilution assay was performed. The cell culture glass tubes were cleaned rigorously before use by physical means utilizing a bottlebrush and detergent (Antiseptica, Germany). This was followed by a chemical cleaning step with 65% nitric acid overnight. Instantly before starting the dilution assay, the overnight cultures were diluted in BHI to an OD_630_ of 0.1. To obtain a stock emulsion of 20 mg/ml, 215 μl of 1.8-Cineol was added to 9.785 ml of BHI in a cell culture glass tube and vortexed vigorously. The 1,8-Cineol stock emulsion was further diluted geometrically to concentrations of 10, 5, 2.5, and 1.25 mg/ml. The total culture volume used was 1 ml per tube. Finally, the tubes were sealed with Parafilm and incubated for 6 h at 37°C at 250 rpm. To determine the MIC, the OD_630_ was measured as biological triplicates.

### Determination of MBIC_50_ of Pure Cultures

To determine the minimal biofilm inhibitory concentration (MBIC_50_) of 1,8-Cineol, a macrodilution assay was performed. The material was prepared in a equal manner as described above for the MIC assay. For the three different germs three different geometric dilutions were prepared from the 20 mg/ml 1,8-Cineol stock emulsion. For *S. aureus* the dilution range was set between 1.25 and 0.075 mg/ml, for *E. coli* the range span the interval between 5 and 0.3 mg/ml and for *M. catarrhalis* it ranged from 10 mg/ml down to 0.625 mg/ml. The overnight cultures were diluted in BHI to an initial inoculum density (OD_630_) of 0.01 for *S. aureus*, 0.2 for *E. coli* and 0.0001 for *M. catarrhalis.* The glass tubes were incubated at 37°C and 250 rpm. After 18 h of incubation the medium was changed to fresh BHI containing the appropriate amount of 1,8-Cineol. After an overall incubation time of 24 h the tubes were washed once with PBS to remove the planktonic cells. The remaining biofilm was stained with a crystal violet solution (0.1% in distilled water; Sigma-Aldrich, United States) for ten minutes and washed three times with distilled water. The dye was dissolved with 1 ml ethanol added to the tubes. The MBIC_50_ was spectroscopically determined at OD_595_ as biological triplicates.

### RT-qPCR

To analyze the cause of biofilm inhibition on the transcriptional level, bacterial cultures were prepared as described in 2.4 with media containing 1 mg/ml 1,8-Cineol. The samples were taken after 2, 6, and 24 h. To derive mRNA from the samples the cell suspension was pelleted and the biofilm was scraped from the walls of the glass tubes both sample types were incubated with 100 μl TE buffer (10 mM Tris, 1 mM EDTA, pH 8) containing 5 mg/ml lysozyme (L6876, Sigma-Aldrich, United States) for 30 min @37°C. Subsequently the cell suspension was mechanical disrupted in a bullet blender (BBX24; Next Advance, United States) using a volume of approximately 100 μl ZiO_2_ beads with a diameter of 0.5 mm (speed set to 8, time set to 3). Finally the RNA was derived from the homogenized cells suspension by a RNA extraction kit (innuPREP DNA/RNA Mini Kit 2.0; Analytik Jena, Germany) and transcribed to cDNA (RevertAid First Strand cDNA Synthesis Kit, Thermo Fisher, United States). RT-qPCR was performed using the magnetic induction cycler (MIC, BMS, Australia) utilizing a ready to use master mix (Luna Universal qPCR Master Mix; NEB, United States) containing 200 nM Primer (Supplementary Table S1) in a 10 μl sample size as technical triplicate. As reference gene for quantification served the 16SrRNA transcript.

To extract the mRNA from the respiratory epithelium co-cultured with a *S. aureus* biofilm with or without treatment with 1 mg/ml 1,8-Cineol, the standard protocol according to the operators manual from the DNA/RNA extraction kit was applied. Further processing was executed as described above for the bacterial RNA (primers listed in Supplementary Table S1) with GAPDH serving as housekeeping gene.

### Determination of MBIC_50_ in the Host Model System

To determine the MBIC_50_ of the biofilm grown on top of respiratory epithelium, a MTT assay was used to measure the metabolic activity. First, the matured epithelium on the cell culture inserts were inoculated with 0.5 ml of an overnight culture diluted to an OD_630_ of 0.1 in PneumaCult^TM^-ALI Medium containing a geometrical dilution of 1,8-Cineol spanning from 1 mg/ml down to 0.0625 mg/ml as biological triplicates. After 2 h, the bacterial cells were attached to the epithelium and the medium was changed to fresh PneumaCult^TM^-ALI Medium comprising the same geometrical dilution of 1,8-Cineol. An alike medium change was executed after 3, 4,and 5 h. After 6 h, the medium in the inserts was changed to RPMI medium without phenol red (Thermo Fischer, United States) supplemented with 0.5 mg/ml Thiazolyl Blue Tetrazolium Bromide (Sigma-Aldrich, United States) and incubated for 20 min at 37°C. After gentle removal of the RPMI medium the formazan crystals were dissolved in 1 ml DMSO. The metabolic activity of the co-culture was measured as difference between the optical density at 550 nm and 690 nm.

In addition, a molecular biology-based approach was applied to measure the MBIC_50_ in the co-culture system. Preparations of the co-culture and media changes during the incubation period were equal to the process described above. After 6 h of incubation, gDNA of viable cells inside the biofilm was extracted. For this purpose, the biofilm was incubated with a solution 250 μM Propidium Monoazide (40013; Promocell, Germany) in PneumaCult^TM^-ALI Medium for 10 min in the dark. Subsequently the samples were placed on aluminum foil on top of ice and illuminated with a 1000 W halogen lamp from 30 cm distance for 5 min. Following this photo reactive step the gDNA was extracted by identical means as described for the bacterial RNA. Except the biofilm was first incubated with Lysozyme, than detached using the RLT lysis buffer to dissolve the underlying epithelium and processed in the bullet blender before the gDNA was extracted by the DNA/RNA extraction kit. The processed gDNA samples were quantified by qPCR using the magnetic induction cycler as described above. The final content of gDNA per sample was determined as mean of three arbitrary genes (spx, SarA and ica C; Supplementary Table S1).

### Electron Microscopic Imaging

To prepare the biofilm on respiratory mucosa, the organotypic slices were incubated with an overnight culture of *S. aureus* diluted to an OD_630_ of 0.1 in PneumaCult^TM^-ALI Medium containing either 0 mg/ml or 1 mg/ml 1,8-Cineol. Media was changed as described in 2.6. After 6 h des media was removed and substituted with a solution of 2% paraformaldehyde (Sigma-Aldrich, United States), 2% glutaraldehyde (electron microscopic grade; Polysciences Inc., United States) and 0.15% alcian blue in PBS and incubated for 22 h at 4°C. After three washing steps with PBS the samples were post fixated with 1% OsO_4_ (Sigma-Aldrich, United States) for 90 min in PBS at room temperature and finally freeze dried. For this purpose the specimens were washed in deionized water and nearly sucked dry, resulting in a very thin film of water. Subsequently, the samples were plunge frozen in liquid nitrogen (the commonly utilized liquefied propane disrupts the biofilm matrix). The frozen samples were transferred at −196°C into a self-made freeze-drying apparatus (mainly consisting out of two heavy polished copper blocks spaced apart by a distance of 5 mm). The copper blocks were brought into a high-vacuum chamber and dried for 17 h at 10^–5^ mbar. Subsequently, the samples were sputter-coated with a continuous layer of 10 nm tantalum and imaged by the Helios Nanolab 600 (FEI, United States) in secondary electron mode at 5 kV.

### Confocal Imaging

For confocal microscopy the biofilm on top of the organotypic slices was prepared as described in 2.7. After 6 h, the slices were fixed in 4% paraformaldehyde for 30 min and washed in PBS. The staining of DNA was accomplished by submerging the sample for 10 min in a solution of 1 μg/ml DAPI (Sigma-Aldrich, United States). To stain eukaryotic F-actins the slices were incubated for 20 min in PBS containing 4 units/ml Phalloidin coupled to Alexa Fluor 647 (Promocell, Germany). The slices were cut perpendicular to the epithelial layer in smaller pieces and prepared as hole mounts in mowiol between a microscopic slide and a cover slip separated by a 300 μm spacer. Imaging was performed with a confocal laser scanning microscope (CLSM; CLSM 780 Carl Zeiss, Germany).

## Results

### Comparison of Microbiological Colonization Between Healthy Nasal Cavities and CRS Patients

**FIGURE 1:**
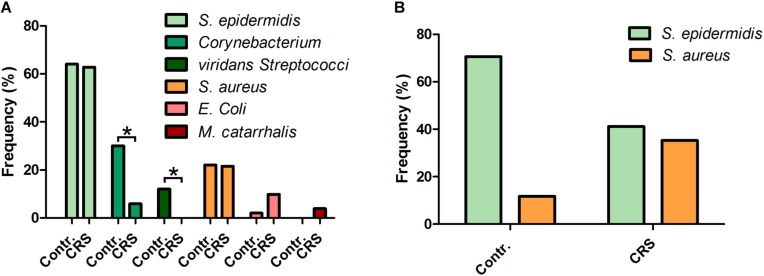
The normal microbiome compared to the microbiome of CRS patients. **(A)** MALDI-TOF-MS-based analysis of the microbiome of CRS patients and a healthy control group with clinical standardized pre-culture (*n* = 50). The commensal Corynebacterium and viridans Streptococci species are more frequent in healthy sinuses, while potential pathogenic bacteria like *E. coli* and *M. catarrhalis* are more often detectable in CRS patients. **(B)** MALDI-TOF-MS-based analysis of the microbiomes with pre-cultivation on Baird-Parker agar. The commensal bacterium *S. epidermidis* occurs less frequent in CRS patients at the same time the potential pathogen *S. aureus* can be detected more often (^∗^≤0.05; binomial test; one-tailed).

To gain an overview regarding the most prominent germs located in nasal cavities of CRS patients, a microbiological analysis of clinical grade quality was executed on 50 CRS patients treated at the general Hospital of Bielefeld, Germany. To obtain better insights into the microbiome shift in our test group, we additionally recruited 50 patients treated for diseases not related to inflamed sinuses from the same demographic group and analyzed their nasal swaps in the same manner as for the CRS patients (Figure 1A).

Our results show that the microbiome of the test group is shifted from commensal bacteria genuses like Corynebacterium and viridans Streptococci to the potential pathogenic species *E. coli* and *M. catarrhalis* (for listing of all germs detected in every single test person cf. Supplementary Figure S1). The frequency of the commensal genus was significantly reduced from 30 to 6% (Corynebacterium) and from 12 to 0% (viridans Streptococci). The two pathogenic germs *E. coli* and *M. catarrhalis* where found with a frequency of 10 and 4% respectively in CRS patients and only 2 and 0% respectively of the healthy test individuals were colonized by this germs. Even though we found the bacteria *S. aureus* in about 20% of all CRS patients we could not verify the high prevalence expected. An additional microbiological analysis of the same demographic group with a pre-cultivation step optimized for the detection of Staphylococci species revealed a frequency of 36% for *S. aureus* in CRS patients and only 12% in the healthy control group (Figure 1B). This was accompanied by a reduced presence of *S. epidermidis* in CRS patients compared to healthy individuals of the same demographic group.

**FIGURE 2:**
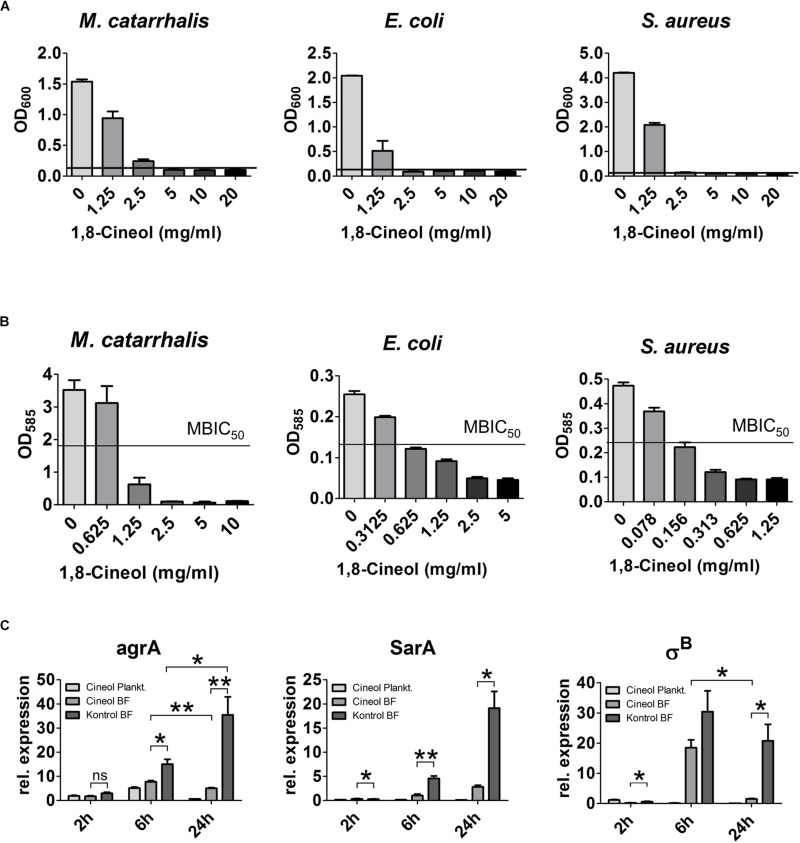
The antibacterial properties of 1,8-Cineol on different CRS related pathogens. **(A)** The MIC was determined for *E. coli*, *M. catarrhalis* and *S. aureus* after 6 h of incubation with the indicated concentration of 1,8-Cineol. The bacteria *M. catarrhalis* was the least susceptible exhibiting a MIC of 5 mg/ml also *S. aureus* showed a slight growth at 2.5 mg/ml only the growth of *E. coli* was completely inhibited by a concentration of 2.5 mg/ml 1,8-Cineol. **(B)** The MBIC_50_ was measured for the pure culture of all three different bacterial strains. The germs *M. catarrhalis* and *E. coli* showed the smallest inhibition of biofilm growth upon exposure to 1,8-Cineol with MBIC_50_ values between 1.25 mg/ml and 0.625 mg/ml and around 0.625 mg/ml, respectively. *S. aureus* showed a high sensitivity in terms of biofilm growth with a MBIC_50_ of only 0.15 mg/ml. **(C)** RT-qPCR analysis of the transcripts of the major key players in biofilm generation of *S. aureus* upon treatment with 1 mg/ml 1,8-Cineol normalized to the initial expression level of the inoculum. All three key players are downregulated upon treatment with 1,8-Cineol. After 2 h only subtle differences are detectable; after 6 h of incubation the expression level in sessile and planktonic bacteria treated with 1,8-Cineol is already significantly reduced. After 24h of biofilm maturation this difference becomes much more prominent and the corresponding expression levels of 1,8-Cineol treated sessile bacteria make up only a fraction of the non-treated ones (^∗∗^ ≤ 0.01, ^∗^ ≤ 0.05, ns > 0.05; unpaired *t*-test; one-tailed, confidence interval: 95%).

To test the antimicrobial properties of 1,8-Cineol on the most prominent pathogens detected in CRS patients we performed MIC and MBIC_50_ assays on pure cultures of *M. catarrhalis*, *E. coli* and *S. aureus* (Figures 2A,B). All three germs showed a similarly high reduced rate of growth in their planktonic live form. The corresponding MIC values laid between 2.5 and 5 mg/ml for *S. aureus* and *M. catarrhalis.* But it can be estimated that the MIC value for *S. aureus* was closer to 2.5 than 5 mg/ml, since this germ showed only a slight growth at 2.5 mg/ml to an OD_630_ of 0.138. The MIC value for *E. coli* could be circumscribed to the interval between 2.5 and 1.25 mg/ml. As it is highly significant for the treatment of the biofilm-based CRS disease, we determined the inhibition of biofilm growth induced by 1,8-Cineol. The MBIC_50_ for the germs *M. catarrhalis* and *E. coli* were rather high and lay between 1.25 and 0.625 mg/ml or around 0.625 mg/ml, respectively. Contrary to that, *S. aureus* showed an extraordinary strong inhibition of biofilm growth as demonstrated by a low MBIC_50_ of around 0.15 mg/ml. When we define a reasonable reduction of the inhibitory effect as an upregulation of growth by at least a factor of two, we see that this occurs at 1,8-Cineol concentration of around 1.25 mg/ml for the germs *M. catarrhalis* and *E. coli* independent from their live form (sessile or planktonic). Considering this definition of a reasonable inhibitory effect, for the germ *S. aureus* we measured a concentration of 1.25 mg/ml for the planktonic and 0.156 mg/ml in case of the sessile live form. Since these data cannot be simply explained by a reduced amount of biomass due to reduction of growth, we performed RT-qPCR based experiments to gain deeper insight into this strong biofilm inhibiting effect (Figure 2C). Hence we quantified the expression of mayor key players in biofilm development, growth, and maturation in *S. aureus*, namely SarA, agrA and σ^B^. After 2h, all transcripts were heavily downregulated in comparison to the expression level of the planktonic *S. aureus* of the inoculum. The ratio ranged between only 0.2% for σ^B^ in the biofilm treated with 1,8-Cineol and 3% for agrA in the untreated biofilm. The expression of σ^B^ and SarA in the biofilm treated with 1,8-Cineol was significantly lower, but only subtly decreased in comparison to the untreated ones. After 6 h of cultivation however, we found a strong and significant downregulation of the transcripts of agrA and SarA in biofilms treated with 1,8-Cineol. At the same time the transcript of σ^B^ was heavily upregulated in both biofilm samples. Even though σ^B^ was less upregulated in the treated biofilm this effect was not significant. When the difference between the treated and untreated biofilms after 24 h is considered the downregulation of the transcripts of agrA, SarA and σ^B^ by the treatment is much more pronounced (agrA: 7 fold, SarA: 6.75 fold and σ^B^:13.78 fold). Remarkably, when the changes in transcription in the treated biofilm during its maturation between 6 and 24 h of culture are compared, the transcripts of agrA and σ^B^ were actually significantly downregulated (agrA: 1.53 fold and σ^B^:12.45 fold). As opposed to this, the transcript of agrA was significantly upregulated by a factor of 2.36 and that of σ^B^ insignificantly and only slightly decreased at the very most in the untreated biofilm during the same time span.

The electron microscopic investigation of treated and untreated *S. aureus* biofilm sowed a change in composition to a higher concentration of aldehyde fixable substances (cf. Supplementary Figure S2).

**FIGURE 3:**
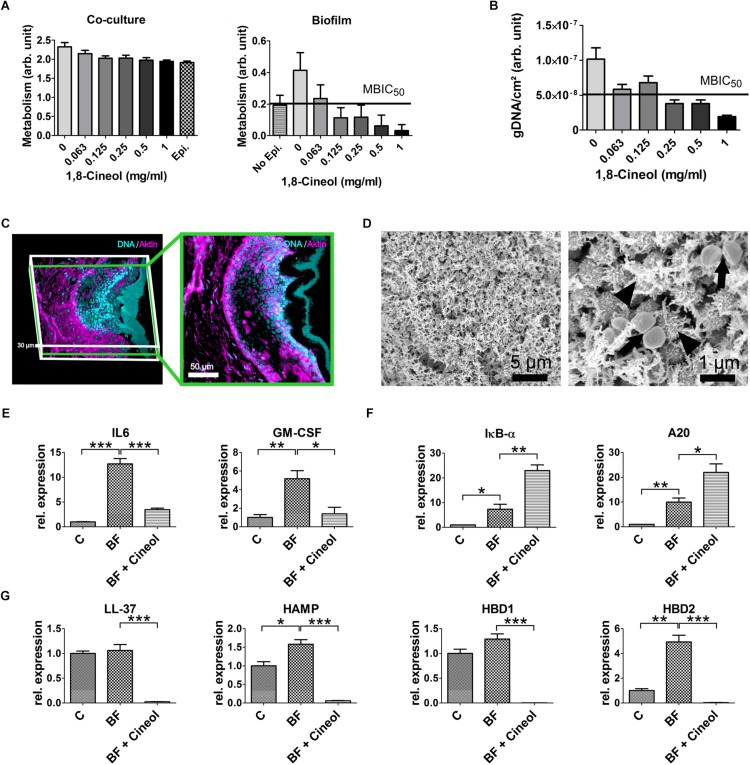
Generation of a *S. aureus* biofilm and its effect on the *in vitro* host models of CRS. **(A)** Determination of the MBIC_50_ in an *in vitro* host model via a MTT assay. The metabolism of the co-culture is the sum of the metabolism of the respiratory epithelium and the biofilm formed by *S. aureus* (left). When the metabolism of the underlying epithelium (epi.) is subtracted from the metabolism of the co-culture (right), a MBIC_50_ between 0,125 and 0.0625 mg/ml 1,8-Cineol can be derived. The amount of biofilm grown without underlying epithelium (no epi.) is about half as much as with epithelium, emphasizing the biofilm promoting properties of the epithelium. **(B)** A RT-qPCR based approach to measure the MBIC_50_ in the host model. After extraction of gDNA from the viable bacterial cells the measured amount of gDNA lead to an MBIC_50_ around 0.125 mg/ml 1,8-Cineol. **(C)** 3D-reconstruction of confocal laser scanning microscopic data (left) and a cross section (right) showing an organotypic slice co-cultivated with *S. aureus*. The biofilm is solely stained with DAPI (blue) while the eukaryotic actin filaments are also stained with Phalloidin (magenta). The biofilm comprises a thickness between 5 and 15 μm and is separated from the underlying respiratory epithelium by a mucus layer spanning over 10–30 μm. **(D)** Scanning electron microscopic image of a biofilm grown on organotypic slices. The biofilm homogenously covers the respiratory mucosa and consist out of a dense network of EPS embedding the bacterial cells. High resolution images show that some cells secrete these EPS (arrowheads) while other cells are dividing (arrows). **(E)** Comparison of cytokine transcription of the uninfected host model **(C)** to the biofilm infected model (BF) and the reaction upon treatment with 1,8-Cineol (BF + Cineol): IL 6 and GM-CSF were upregulated by the biofilm infection. The treatment with 1,8-Cineol dampened this inflammatory reaction markedly. **(F)** RT-qPCR investigation of target genes of the NF-kB pathway A20 and IKB-α. Both transcripts are upregulated by the biofilm growth. The addition of 1,8-Cineol results in a further upregulation of these two negative regulators. **(G)** RT-qPCR data of the relative expression of antimicrobial peptides showing a slight upregulation upon biofilm growth and a very strong downregulation upon treatment with 1,8-Cineol (^∗∗∗^ ≤ 0.001, ^∗∗^ ≤ 0.01, ^∗^ ≤ 0.05; unpaired *t*-test; one-tailed, confidence interval: 95%).

To analyze the extraordinary high biofilm inhibiting potential of 1,8-Cineol on the germ *S. aureus* in a situation more closely related to the *in vivo* situation we performed different MBIC_50_ measurements in an *in vitro* host model of CRS. The model comprises a mature respiratory epithelium inside a cell culture insert on top of this epithelium a biofilm was grown. The maturation of the epithelium layer was confirmed for three independent donors by markers for the maturation of tight junctions, goblet- and ciliated cells (cf. Supplementary Figure S3). Since a quantification of the biofilm mass by staining was not feasible the amount of grown biofilm was assessed by measuring the metabolic activity of the biofilm on the defined surface area of the insert (Figure 3A). By this, we estimated the MBIC_50_ in our host model system at between 0.0625 and 0.125 mg/ml. Besides this we observed, that the amount of biofilm measured without underlying epithelium was about half as much compared to the biofilm adhered onto the epithelial layer. To verify the measured MBIC_50_, we additionally conducted a determination of the MBIC_50_ based on the quantification of the viable cells in the biofilm. This was done by isolating the gDNA of viable cells on the defined surface area and quantifying this amount by qPCR (Figure 3B). The measured MBIC_50_ laid around 0.12 mg/ml and thus was similar to the one derived from the metabolism based approach but with a smaller standard deviation for the measured biofilm amount.

To determine the effect of the biofilm and its treatment by 1,8-Cineol on the innate immune system, we choose to alter our CRS host model by utilizing organotypic slices overgrown by a biofilm. By this setup, the effect on all cell types residing in the mucosal tissue and responsible for the initial response of the innate immune system was measured. As demonstrated by CLSM, we were able to grow an intact mature biofilm on top of the mucus layer covering the organotypic slice (Figure 3C). Furthermore, the CLSM images exhibited, that the utilized organotypic slices contained the respiratory epithelium as well as the lamina propria. Scanning electron microscopic images of the biofilm showed a dense network of EPS and an overall homogenous morphology (Figure 3D). Interestingly, the high resolution images (Figure 3D, right) allowed distinguishing between cells secreting EPS and proliferating cells. In this model system we detected the significant upregulation of the cytokines IL6 and GM-CSF by a factor 12.69 and 5.18, respectively. To a lesser extent, the antimicrobial peptides HAMP and HBD2 exhibited also a significant upregulation upon biofilm formation (HAMP: 1.58 fold and HBD2: 4.93 fold) (Figures 3E,G). This inflammatory response was driven by the NF-κB pathway as demonstrated by the significant upregulation of NF-κB specific target genes IKB-α and A20 by a factor of 7.34 and 9.98, respectively (Figure 3F). As expected, 1,8-Cineol reduced biofilm formation and hence inflammation leading to an significant reduction of cytokine expression approaching the expression level of the uninfected respiratory mucosa (IL6: 3.52 fold, GM-CSF: 2.49 fold). This anti-inflammatory action was further underlined by the significant upregulation of negative regulators of the NF-κB pathway IKB-α and A20 by a factor 3.12 and 2.2, respectively (Figure 3F). The downregulation of NF-κB target genes was especially notable at the transcriptional level of antibacterial peptides (Figure 3G), which were altogether highly significantly downregulated much below the initial expression level as consequence of the treatment with 1,8-Cineol (LL-37: 44.41 fold, HAMP: 25.84 fold, HBD1: 730.59 fold, HBD2: 155.6 fold; *p* ≤ 0.001).

## Discussion

The results obtained by analyzing the microbiology of our test group display a shift in the microbiome in the sinuses of CRS patients. In general, we detected a decrease in microbiome diversity in CRS patients from 1.7 species per patient to 1.39 species. A similar decrease could be detected in two genomic based studies (Abreu et al., 2012; Choi et al., 2014). In contrast to that another genome based analysis by Aurora et al. detected an increase in diversity in CRS patients (Aurora et al., 2013). Anyhow, it should be considered that the antibiotic usage of CRS patients prior to sampling may reduce the microbiome diversity. In terms of composition, we could detect a decrease of *Corynebacterium* species in our test group, which was also detected by pyrosequencing (Feazel et al., 2012) and culture based (Klossek et al., 1998) approaches. Interestingly, in this investigation the prevalence of streptococcus viridians species decreased in CRS patient down to zero. In contrast to that other solely culture based approaches detected an increase from 3.7 to 10% (Klossek et al., 1998) or frequencies of about 8.3% (Biel et al., 1998) of this species in CRS. According to previous studies (Jefferson et al., 2005; Feazel et al., 2012; Tan et al., 2012; Boase et al., 2013), we detected *S. aureus* with a higher frequency in CRS patients compared to healthy individuals of the same demographic group. The *S. aureus* frequency of about 40% detected in CRS patients in this study is in good accordance to studies applying similar culture based approaches (Biel et al., 1998; Carson et al., 2002; Kavanaugh and Ribbeck, 2012; Honorio et al., 2015) and one fluorescence *in situ* hybridization (FISH) based investigation (Niederfuhr et al., 2008). Other studies reported higher abundancies of *S. aureus* in CRS. They applied sequencing based techniques (Feazel et al., 2012) but also FISH (Nostro et al., 2007). The differences between the two FISH based approaches might be the difference in sampling techniques (one used lavages the other one biopsies). Surprisingly, it seemed that the high frequency of *S. aureus* was masked by the pre-cultivation step on agar media commonly used during clinical diagnosis. We think it is conceivable that even though we optimized the pre-cultivation for staphylococcus species, at least a few of the *S. aureus* present in the sample were not cultivated. This would further elucidate the fundamental drawback of the rather cheap culture based methods applied in clinical practice all over the world. The observed reduction of *S. epidermidis* abundance is in good accordance with a pyrosequencing based study (Feazel et al., 2012). Interestingly, animal (Cleland et al., 2014) and *in vitro* (Iwase et al., 2010) studies demonstrated the ability of *S. epidermidis* to suppress the pathological properties of *S. aureus*. In addition, extensive microbiological examination (*n* = 50) showed that the samples taken from patients with CRS contained *E. coli* and *M. catarrhalis* with a higher frequency compared to the healthy control group. This result is consistent with studies describing biofilms of *E. coli* in CRS patients applying SEM (Perloff and Palmer, 2004) or culture based approaches (Cleland et al., 2013). *M. catarrhalis* was also described as pathogen in CRS in an study based on molecular biological techniques (Boase et al., 2013; Choi et al., 2014). In contradiction to the literature, we were only able to detect the species *Haemophilus influenzae* once and the germ *P. aeruginosa* not at all (Supplementary Figure S1). This is noteworthy since, according to studies based on culture-, FISH- and molecular biological methods (Sanderson et al., 2006; Prince et al., 2008; Foreman et al., 2009; Oncel et al., 2010; Stressmann et al., 2011), this germ is often detectable in CRS. At this point it should be noted that in the study of Sanderson et al. (2006) *P. aeruginosa* couldn’t ’be detected by FISH probes in any of the 18 samples analyzed.

Considering our microbiome data of CRS patients, we executed antimicrobial assays on *E. coli*, *M. catarrhalis* and *S. aureus*. The MIC assays showed that not only *S. aureus*, but also the Gram-negative bacteria *E. coli* and *M. catarrhalis*, responded well to the treatment with 1,8-Cineol. This is remarkable, since most Gram-negative bacteria poorly react to the inhibitory effects of essential oils (Nazzaro et al., 2013). This drug resistance is mainly mediated by the outer membrane, which acts as first line defense against many hydrophobic molecules. Anyhow, some essential oils are not held back by the outer membrane and unfold their inhibitory potential also against Gram-negative bacteria (reviewed in Swamy et al., 2016). In the case of 1,8-Cineol it is conceivable, that, the outer membrane is an insufficient barrier for the small non-polar 1,8-Cineol molecule, which might overcome the membrane through porin molecules (Plesiat and Nikaido, 1992).

The experimental determination of the inhibitory effect of 1,8-Cineol on biofilm formation showed a correlative effect between inhibition of growth in the planktonic and sessile form of *E. coli* and *M. catarrhalis*. Remarkably, the bacterium *S. aureus* reacts with a high sensitivity toward 1,8-Cineol, with a susceptibility not explainable by a plain inhibition of bacterial growth. Further investigation regarding the action of 1,8-Cineol on the key players of the biofilm formation pathways in *S. aureus* elucidated a strong inhibitory effect on the transcription of agrA, SarA and σ^B^ gens. SEM images of the treated and untreated biofilm of *S. aureus* showed a biofilm which was more susceptible to the chemical fixation applied in sample preparation for SEM. We assume that the stress induced changes in the expression pattern lead to a higher concentration of aldehyde fixable substances. proteins e.g., amyloid fibers and deacetylated PIA. Thus, we conclude that 1,8-Cineol disrupts the intracellular signaling pathways essential for biofilm formation. The multimodal disturbance on all three key regulators is of particular interest, since this will prevent the development of sessile bacteria resistant against all modes of action of 1,8-Cineol. This is of particular interest, since the common antibiotics used to treat purulent CRS acting on just one key player, making them vulnerable to the development of antibiotic tolerances. Even worth the common antibiotics are not able to efficiently penetrate the biofilm. Hence, a gradient of antibiotics is established inside of the biofilm (Jefferson et al., 2005). The resulting exposure of the bacteria to sub lethal dosages will furthermore promote the development of antibiotic tolerances in CRS. Since 1,8-Cineol penetrates the biofilm much more efficiently, this path to bacterial resistances is also blocked by 1,8-Cineol. To further investigate this pharmacological action of 1,8-Cineol in a scenario more closely related to the actual *in vivo* situation, we developed two different host model systems. The first one is comprised of a co-culture system of maturated respiratory epithelium at the air-liquid interface colonized by a *S. aureus* biofilm. The defined surface area size of the respiratory epithelium in the cell culture insert enabled the 1,8-Cineol MBIC_50_ measurement by determining the amount of biofilm grown in each insert. Since the epithelium and the mucus layer covering the maturated epithelium in the inserts is the only substance in direct physical contact to the adhering bacteria (Bosch et al., 2013) this model is sufficient to simulate the enhanced bacterial adhesion and biofilm growth in the sinuses. This way, we were able to determine an MBIC_50_ of 1,8-Cineol for *S. aureus* between 0,125 and 0,0625 mg/ml. We measured the MBIC_50_ values with two independent methods. One is based on the metabolic activity in the co-culture system and the other one determines the quantity of vital bacteria via their genomic DNA. It is important to mention, that the standard deviation of the measured biofilm amount is smaller when the second approach was applied. This might be due to the high metabolic background of the respiratory epithelium in the co-culture system. It is conceivable, that this might be improved by fixating the respiratory epithelium prior to the experiment with formalin. But this would alter the surface properties e.g., receptors for bacterial adhesion and hence would render the approach les close to the *in vivo* situation. Hence we would suggest to apply the second method in further studies. The second model system consists of an organotypic slice overgrown by a *S. aureus* biofilm. This model is appropriate to measure the impact of the biofilm and the effect of pharmaceutical treatments on the whole respiratory mucosa. We demonstrated that a mature biofilm could be formed in this model system and that the organotypic slices contained the epithelium as well as the lamina propria. In this setup, the pathogen-associated molecular patterns derived from the bacteria are able to act on the toll-like receptors of the cell population of the first line defense system in mucosal tissue. We used this model to study the innate immune response upon biofilm formation more exact the expression of cytokines and antimicrobial peptides known to be secreted by the respiratory mucosa (reviewed in Parker and Prince, 2011). In accordance to the literature (reviewed in Kawai and Akira, 2006), NF-kB is at least partly responsible for the transcription of cytokines and antimicrobial peptides (Frohm et al., 1997; Tsutsumi-Ishii and Nagaoka, 2002; Frazier et al., 2011). Beyond this, we could demonstrate that the pharmacological action of 1,8-Cineol was not restricted to the inhibition of biofilm growth alone. In fact, we could detect an upregulation of the transcripts of the negative regulators A20 and IKB-α of NF-kB signaling resulting in a further dampening of the inflammatory reaction. Even though this was accompanied by a striking downregulation of antimicrobial peptides, we do not consider this as a drawback. Since the antimicrobial peptides are known to poorly penetrate deep into the biofilm (Otto, 2006) which annihilates their antimicrobial potential in a biofilm environment anyhow.

## Conclusion

In conclusion, this study has shown that *S. aureus* is the dominant germ in patients with CRS. A promising finding is the good biofilm-inhibiting effect of 1,8-Cineol on this organism. Especially because, its mode of action is unfolded on all key players of the biofilm generation pathway, thereby reducing the chance of resistance development. In a 1,8-Cineol-based biofilm therapy, the high diffusion potential of 1,8-Cineol would also be an outstanding advantage in terms of agent accessibility to bacteria and the development of resistances due to agent gradient. This all-new treatment of bacterial biofilm infections could find application beyond CRS in many biofilm-based infectious diseases. For example, the common biofilm colonization of catheters with *S. epidermidis* is regulated by a very similar biofilm generation pathway as described in *S. aureus* (O’Gara, 2007). Anyhow, most biofilm infection might be based on a complex microbiome composed out of many species. Thus, a further analysis on these more complex cultures might give deeper insight on the treatment of biofilm based diseases by 1,8-Cineol. We suggest that the technical innovation of the two presented host model systems can be implemented to model various infection scenarios of the respiratory tract. Depending on the specific requirements of the respective experiment, a defined surface area or a complex organotypic culture can be infected with any biofilm forming organism or microbiome.

## DATA AVAILABILITY STATEMENT

The datasets generated for this study are available on request to the corresponding author.

## ETHICS STATEMENT

Specimens were obtained after informed written consent according to local and international guidelines (Bezirksregierung Detmold/Münster/Ethical Cl. number 2012-015-f-S) and the Declaration of Helsinki.

## AUTHOR CONTRIBUTIONS

MS: conception of the work, the acquisition, analysis, and interpretation of the data, and writing and revising the manuscript. FO: analysis and interpretation of the data and revising the manuscript. MG: acquisition and analysis of the data and revising the manuscript. CJ, CKn, and AH: interpretation of the data and revising the manuscript. CKa and BK: conception of the work and revising the manuscript. HS: conception of the work, analysis and interpretation of the data, and revising the manuscript. BB: acquisition and revising the manuscript.

## Conflict of Interest

The authors declare that the research was conducted in the absence of any commercial or financial relationships that could be construed as a potential conflict of interest.
