# Supplementary material for: The Therapeutic Effect of 1,8-Cineol on Pathogenic Bacteria Species Present in Chronic Rhinosinusitis
Source: Front Microbiol. 2019 Oct 22;10:2325. doi: 10.3389/fmicb.2019.02325 (PMC6821979; doi:10.3389/fmicb.2019.02325)
Supplement: Supplementary file 1 [file Data_Sheet_1.docx]

Supplementary Material

Supplementary Figures
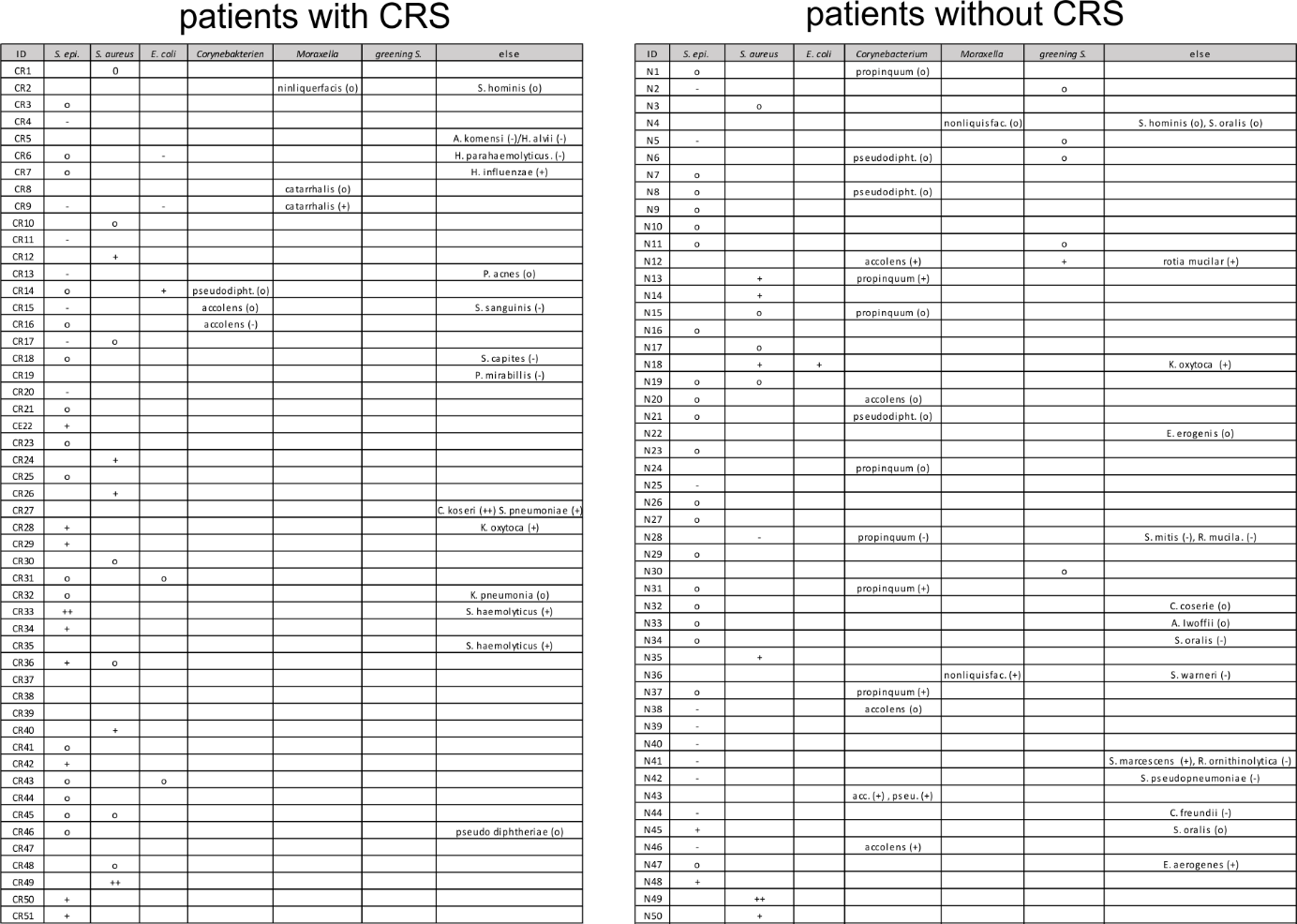


***Fig. S1: Detailed overview of the microbiome analysis shown in Fig. 1 A.*** *The amount of cultured bacteria was classified by the executing microbiologist (- = scattered, o = sparsely, + = common, ++ = abundant)*

*
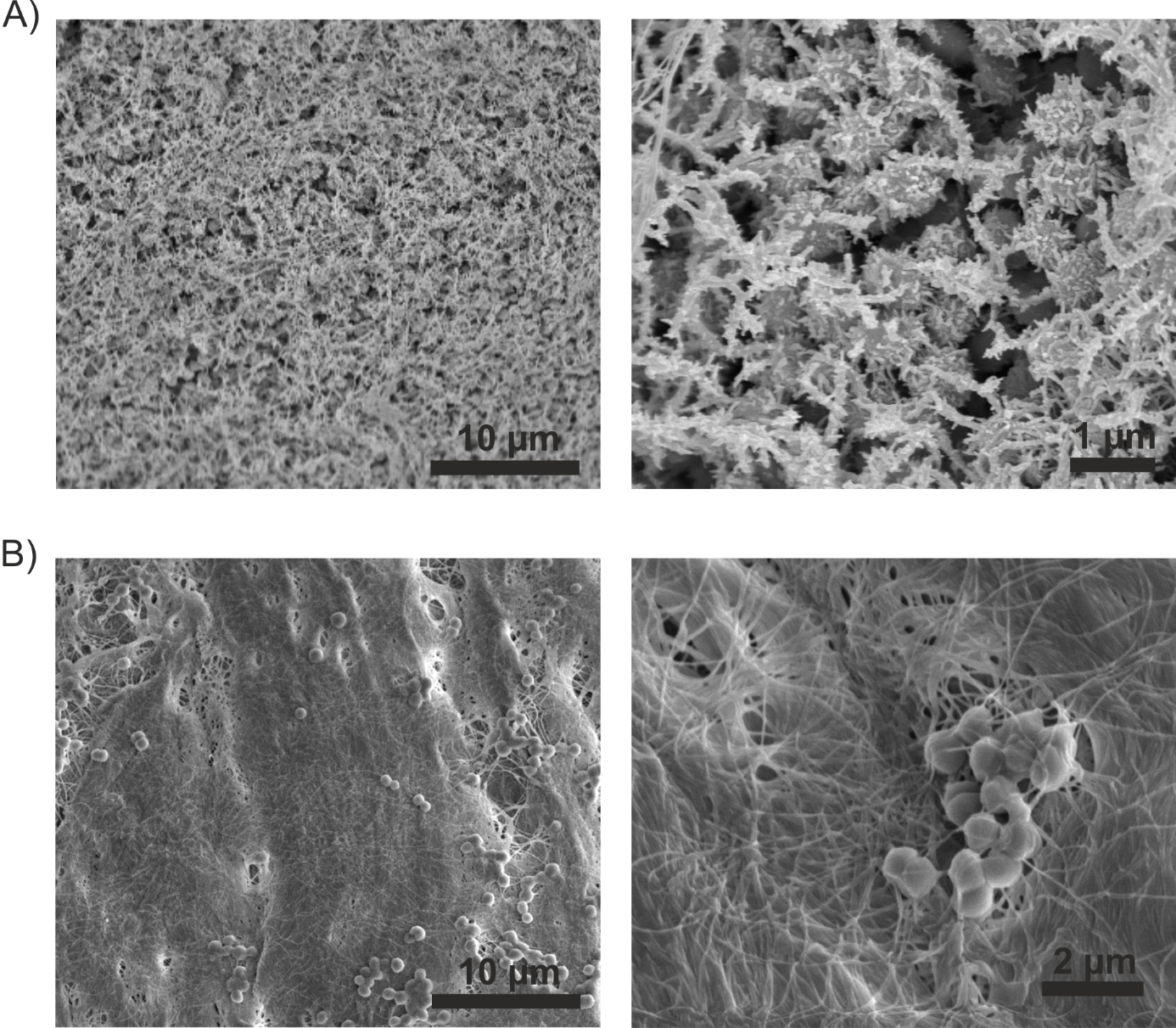
*

***Fig. S2: Effect of 1,8-Cineol on the composition of the S. aureus biofilm imaged by SEM.*** *(A) The untreated biofilms exhibits a less smooth surface at lower magnification (left). At higher magnification (right) it becomes visible, that the single cells are decorated with fixable fibers. (B) The treated biofilm exhibits a different biofilm composition mainly assembled out of fixable substances resulting in a smooth surface in the SEM image (left). At a higher magnification (right) it becomes visible, that the singles cells are encased inside the biofilm matrix which is interweaved by a network of fibers.*


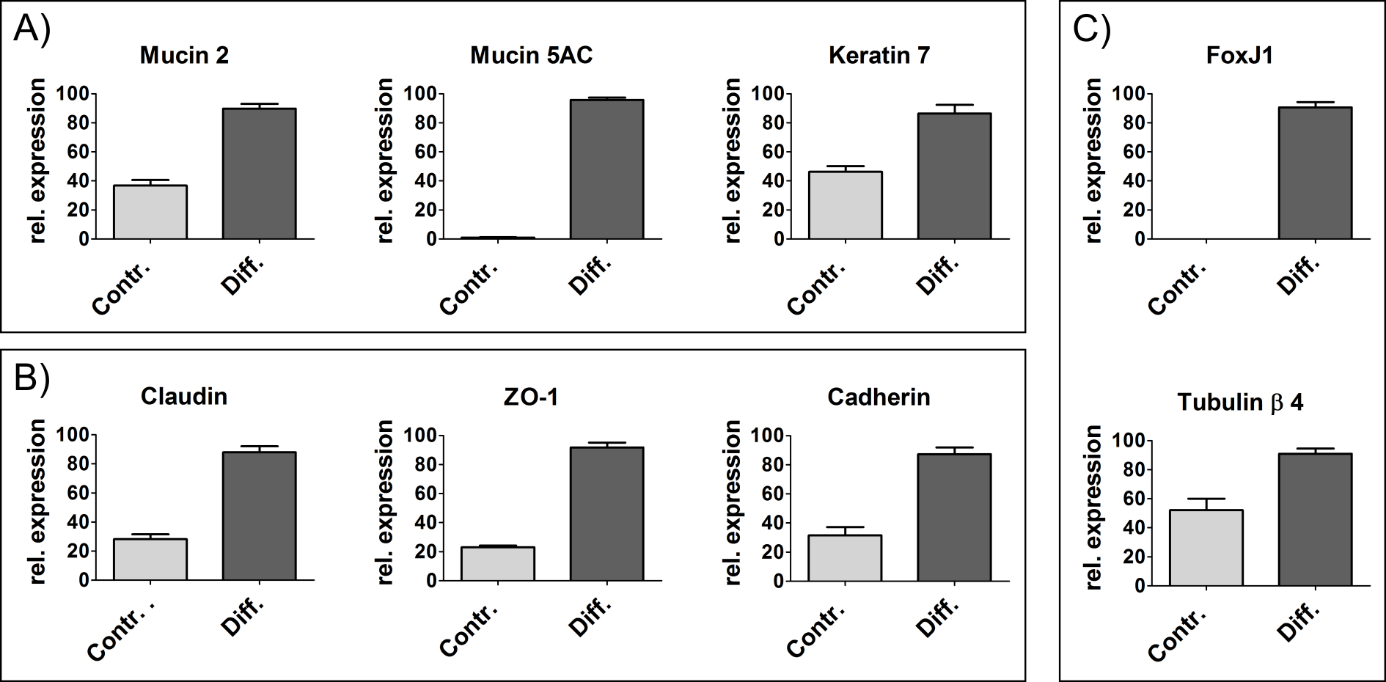


***Fig. S3: RT-qPCR quantification of differentiation markers during maturation of the respiratory mucosa.*** *(A) shows upregulation of markers for gobbled cell differentiation; (B) maturation of the tight junction lead to an upregulation of important intercellular adhesion molecules; (C) the main transcription factor for generation of cilia (FoxJ1) is switched on during mucosa maturation resulting in an upregulation of tubulin β 4 (N=3).*

| **human primer** |  |  | **S. aureus primer** | |
| --- | --- | --- | --- | --- |
| IL6 fw | GCAAAGAGGCACTGGCAGAAAACA |  | Spx fw | CCTGGCTTATTACGTCGTCCA |
| IL6 rev | TTCTGCAGGAACTGGATCAGGACT |  | Spx rev | AACGTCGAATCTCGTCCTCA |
| GM-CSF fw | CAGCCTCACCAAGCTCAAG |  | SarA fw | ACAACCACAAGTTGTTAAAGCAG |
| GM-CSF rev | GGGATGACTGGCAGAAAGTCC |  | SarA rev | ACAACCACAAGTTGTTAAAGCAG |
| IκB-α fw | AGACCTGGCCTTCCTCAACT |  | icaC fw | TGCGTTAGCAAATGGAGACT |
| IκB-α rev | GTCTCGGAGCTCAGGATCAC |  | icaC rev | TGCGTGCAAATACCCAAGAT |
| A20 fw | TACCCTTGGTGACCCTGAAG |  | agrA fw | GAAATTGCCCTCGCAACTGA |
| A20 rev | CCTTGGACGGGGATTTCTAT |  | agrA rev | TGTTACCAACTGGGTCATGCT |
| LL-37 fw | CCTGATGCAAAAGCCCAACC |  | sigma B fw | GCAGACGAAAGTAAGTTCGATCAAT |
| LL-37 rev | CTAAAGCAAACCCCAGCCCA |  | sigma B rev | ACCAATCACTTTATCTT |
| HAMP fw | AAATGCAGATGGGGAAGTGGG |  | 16S rRNA fw | CCAGACTCCTACGGGAGGCAG |
| HAMP rev | ATGCTAAGGCCGGTTCCCT |  | 16S rRNA rev | CGTATTACCGCGGCTGCTG |
| HBD1 fw | CTTCTGCTCACTCCCAGCTC |  |  |  |
| HBD1 rev | TCTGCCTGCCCGATCTTTAC |  |  |  |
| HBD2 fw | GAAGGCTTGATGTCCTCCCC |  |  |  |
| HBD2 rev | AAGGCAGGTAACAGGATCGC |  |  |  |
| GAPDH fw | CTGCACCACCAACTGCTTAG |  |  |  |
| GAPDH rev | GTCTTCTGGGTGGCAGTGAT |  |  |  |

***Table S1: PCR primer*.**

**
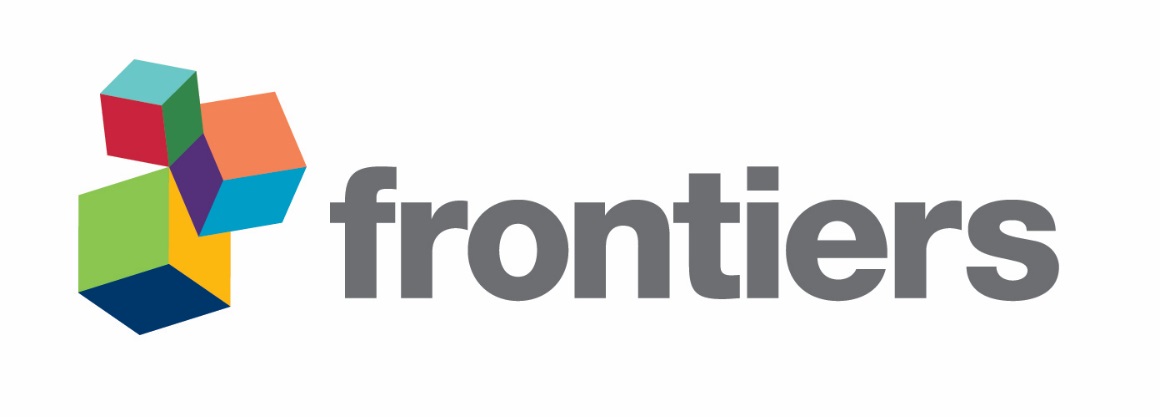
**

**Supplementary Figure 1.** The figure legends are required to have the same font as the main text, 12 point normal Times New Roman, single spaced. Please use a single paragraph for each legend and prepare the figures keeping in mind the PDF layout.
